# The RBP-Jκ Binding Sites within the RTA Promoter Regulate KSHV Latent Infection and Cell Proliferation

**DOI:** 10.1371/journal.ppat.1002479

**Published:** 2012-01-12

**Authors:** Jie Lu, Subhash C. Verma, Qiliang Cai, Abhik Saha, Richard Kuo Dzeng, Erle S. Robertson

**Affiliations:** 1 Department of Microbiology and Tumor Virology Program of the Abramson Cancer Center, Perelman School of Medicine at the University of Pennsylvania, Philadelphia, Pennsylvania, United States of America; 2 Department of Microbiology and Immunology, School of Medicine, University of Nevada, Reno, Nevada, United States of America; University of North Carolina at Chapel Hill, United States of America

## Abstract

Kaposi's sarcoma-associated herpesvirus (KSHV) is tightly linked to at least two lymphoproliferative disorders, primary effusion lymphoma (PEL) and multicentric Castleman's disease (MCD). However, the development of KSHV-mediated lymphoproliferative disease is not fully understood. Here, we generated two recombinant KSHV viruses deleted for the first RBP-Jκ binding site (RTA_1st_) and all three RBP-Jκ binding sites (RTA_all_) within the RTA promoter. Our results showed that RTA_1st_ and RTA_all_ recombinant viruses possess increased viral latency and a decreased capability for lytic replication in HEK 293 cells, enhancing colony formation and proliferation of infected cells. Furthermore, recombinant RTA_1st_ and RTA_all_ viruses showed greater infectivity in human peripheral blood mononuclear cells (PBMCs) relative to wt KSHV. Interestingly, KSHV BAC36 wt, RTA_1st_ and RTA_all_ recombinant viruses infected both T and B cells and all three viruses efficiently infected T and B cells in a time-dependent manner early after infection. Also, the capability of both RTA_1st_ and RTA_all_ recombinant viruses to infect CD19+ B cells was significantly enhanced. Surprisingly, RTA_1st_ and RTA_all_ recombinant viruses showed greater infectivity for CD3+ T cells up to 7 days. Furthermore, studies in Telomerase-immortalized human umbilical vein endothelial (TIVE) cells infected with KSHV corroborated our data that RTA_1st_ and RTA_all_ recombinant viruses have enhanced ability to persist in latently infected cells with increased proliferation. These recombinant viruses now provide a model to explore early stages of primary infection in human PBMCs and development of KSHV-associated lymphoproliferative diseases.

## Author Summary

Kaposi's sarcoma-associated herpesvirus (KSHV) is tightly linked to at least two lymphoproliferative disorders, primary effusion lymphoma (PEL) and multicentric Castleman's disease (MCD). The life cycle of KSHV consists of latent and lytic phase. RTA is the master switch for viral lytic replication. In this study, we first show that recombinant viruses deleted for the RBP-Jκ sites within the RTA promoter have a decreased capability for lytic replication, and thus enhanced colony formation and proliferation of infected cells. Interestingly, the recombinant viruses show greater infectivity in human peripheral blood mononuclear cells (PBMCs). The recombinant viruses also infected CD19+ B cells and CD3+ T cells with increased efficiency in a time-dependent manner and now provide a model which can be used to explore the early stages of primary infection in human PBMCs, as well as the development of KSHV-associated lymphoproliferative diseases.

## Introduction

Kaposi sarcoma-associated herpesvirus (KSHV, also known as human herpesvirus 8 [HHV8]) infection is pivotal to the development of Kaposi sarcoma (KS). KSHV is also strongly associated with two lymphoproliferative diseases, primary effusion lymphoma (PEL) and Multicentric Castleman's disease (MCD) [1], [2]. During its lifespan, KSHV undergoes latent and lytic cycle replication (reactivation). In comparison to lytic cycle replication, fewer genes are expressed in latent infection and a number of these genes are involved in disruption of the cell cycle, and in maintenance of the viral genome. One of those latent genes is Latency-associated nuclear antigen (LANA), encoded by KSHV open reading frame 73 (ORF73), which is critical for persistence of the viral episome and maintenance of latent infection in KSHV infected cells [3]. During lytic cycle replication, almost all viral genes are expressed in a staged temporal manner. The replication and transcription activator (RTA) is encoded by KSHV ORF50 and plays an essential role in the control of the lytic replication cycle. RTA can activate KSHV lytic genes including ORF6 (single-stranded DNA-binding, SSB), ORF21 (thymidine kinase, TS), ORF57 (mRNA transcript accumulation. MTA), ORF59 (polymerase processivity factor, PF-8), ORF 74 (vGPCR), K2 (vIL-6), K5 (MIR-2), K6 (vMIP-1), K8 (k-bZIP), K9 (vIRF), K12 (kaposin), K14(vOX-2) and polyadenylated nuclear (PAN) through direct binding with high affinity to RTA-responsive elements (RREs) or in combination with cellular transcription factors, RBP-Jκ, Ap-1, C/EBP-α, Oct-1, and Sp1[4], [5], [6], [7], [8], [9], [10], [11], [12], [13], [14], [15], [16], [17], [18], [19], [20], [21], [22]. Recombinant viruses that lack RTA establish latency quite efficiently but are unable to reactivate [23]. Our earlier studies also suggest that RTA contributes to the establishment of KSHV latency by activating LANA expression during the early stages of infection through the major effector of the Notch signaling pathway, recombination signal binding protein Jκ (RBP-Jκ). This mutual RTA/LANA feedback regulatory mechanism is likely to be a key event in establishment of KSHV latency and is yet to be completely elucidated.

RBP-Jκ, also named CBF1 or CSL, is a member of the CSL family (CBF1, Suppressor of Hairless, and Lag) and is the major downstream effector of the Notch signaling pathway [24], [25]. RBP-Jκ functions on the target gene by recruiting distinct protein complexes to the promoter. RTA mimics Notch signaling and can activate target promoters by binding to the repression domain of RBP-Jκ, thereby activating promoters [17]. In KSHV-infected cells, RBP-Jκ mediates cooperative transactivation of KSHV genes, ORF57, K-bZIP, ORF6 (SSB), K14 (vGPCR), LANA, K8, ORF47 and RTA [12], [15], [17], [26], [27], [28], [29]. The KSHV RTA promoter contains four potential RBP-Jκ binding sites [30] and mutation of the first and third RBP-Jκ sites within the KSHV RTA promoter results in approximately 50% repression of RTA expression *in vitro* [30].

Understanding the pathogenesis of specific concerns including Kaposi's sarcoma has been aided by development of model systems [31]. It has been difficult to establish lymphoblastoid cell lines in culture by KSHV, which has slowed the understanding of the natural mechanism of KSHV-mediated lymphoproliferative disease. However, the mechanism of differentiation and proliferation due to EBV infection in B lymphocytes are well documented [32]. Recently, two groups have shown that KSHV infects a subset of tonsillar B cells driving plasmablast differentiation and proliferation, and KSHV-encoded viral FLICE-inhibitory protein (vFLIP) induces B lymphocytes transdifferentiation and tumorigenesis in an animal model [33], [34]. Furthermore, Myoung and Ganem reported that T and B lymphocytes in primary human tonsils can be infected by KSHV, with B lymphocytes producing a substantial amount of infectious virions [35], [36]. In contrast to EBV, transformation and immortalization have not been clearly observed in KSHV infected B or T cells. KSHV infection of peripheral blood mononuclear cells (PBMCs) occurs prior to the onset of KS [37], and KSHV DNA is frequently detected in immune-deficient patients [38], [39], [40], [41]. In addition, PBMCs of KSHV infected marmosets support viral infection and replication [42]. Finally, we recently showed that PBMCs exposed to virions from BAC36-293 cells can effectively model early infection [43].

Our previous studies showed that mutation of the RTA promoter in vitro (first and all three RBP-Jκ binding sites) led to significant decreases in RTA activity [30]. RTA is an immediate early protein that serves as the master switch for viral lytic replication. The interaction between RTA and RBP-Jκ controls the activation of multiple viral target genes which are absolutely critical for virus reactivation [17], [26], [27]. Based on these observations, we generated two subtly mutated recombinant KSHV viruses based on BAC36 (wt), RTA_1st_ (deletion of the first RBP-Jκ binding site in the RTA promoter), and RTA_all_ (deletion of all three RBP-Jκ binding sites in the RTA promoter). Our results show that RTA_1st_ and RTA_all_ recombinant viruses had enhanced ability to maintain viral latency and decreased capability to drive lytic replication in infected cells. This resulted in an increase in cell growth and proliferation. Furthermore, RTA_1st_ and RTA_all_ recombinant viruses were enhanced in their ability to infect human PBMCs. Both recombinant viruses infected T and B cells during primary infection, resulting in increased infectivity during the early stage of infection. Long-term persistence of the viral episome in infected cells further confirmed that RTA_1st_ and RTA_all_ recombinant viruses had a greater ability to maintain latent infection and enhanced proliferation. Our study provides specific insights into the contribution of KSHV to its associated lymphomas.

## Materials and Methods

### Cells and plasmids

Human embryonic kidney 293 (HEK 293) cells were maintained in Dulbecco's modified Eagle medium (DMEM) supplemented with 5% bovine growth serum. De-indentified Human peripheral blood mononuclear cells (PBMCs) were obtained from the University of Pennsylvania CFAR Immunology Core. The Core maintains an IRB approved protocol in which Declaration of Helsinki protocols were followed and each donor gave written, informed consent. Telomerase-immortalized human umbilical vein endothelial (TIVE) cells were a kind gift from Dr. Rolf Renne [44]. The wild-type KSHV BACmid, BAC36 wt, was provided by S. J. Gao (University of Texas, San Antonio, TX). Kanamycin (Kn) cassette containing plasmid, pL452 was obtained from the National Cancer Institute Biological Resources Branch.

### Construction of KSHV mutants with in RTA promoter, BAC36-RTA_1st_ and BAC36 RTA_all_ within the RTA promoter

Mutagenesis of BAC36 was performed using the Red Recombination method as described previously [45]. The primers used were BAC 36-RTA_1st_, the forward PCR primer 5′- caaaactgtgtttagtagcaacacaccctggcgagcccagctgtcgaggcCAATTCCGATCATATTCAATAACCCTTAAT -3′ (target sequence is low-cased), and the reverse primer, 5′- tttgagaagcatctttagagagctagaggcttccgtccccaatttcagtaAGAACTAGTGGATCCCCTCGAGGGACCTA -3′ were used to amplify a Kan resistance cassette (underlined sequence is Kan cassette primer) flanked by BAC sequences (genomic position 69057 and 69171, NC_009333); BAC 36-RTA_all_, the forward PCR primer 5′- caaaactgtgtttagtagcaacacaccctggcgagcccagctgtcgaggcCAATTCCGATCATATTCAATAACCCTTAAT -3′ (target sequence is low-cased), and the reverse primer, 5′- atggcgacgtgcactactcgggacccccgcgcaccccggcatatggagtaAGAACTAGTGGATCCCCTCGAGGGACCTA -3′ were used to amplify a Kan resistance cassette (low-cased sequence is Kan cassette primer) flanked by BAC sequences (genomic position 69057 and 70417, NC_009333). The Kan cassette flanked with 50bp KSHV genomic sequence was electroporated into EL350 containing BAC36 at 1.75kV, and the resulting colonies plated on Kanamycin (50 µg/ml) and chloramphenicol (25 µg/ml) double selection plates followed by incubation at 30°C. BAC plasmid DNA was isolated from 10-ml overnight cultures by the alkaline lysis procedure and characterized by restriction enzyme analysis followed by Southern blot analysis. The transformed single colonies were induced with 10% L (+) arabinose (Sigma-Aldrich, Inc., St. Louis, MO) for 1h, then plated to Chloramphenicol and Kanamycin selection plates, separately. The resulting single colonies on Chloramphenicol plates were inoculated in 10-ml of LB with chloramphenicol overnight at 30°C. Small-scale DNA isolation was performed to characterize the DNA of specific mutants, followed by Southern blot analysis. All the clones were confirmed by DNA sequencing using the University of Pennsylvania Perelman School of Medicine sequencing core. Large preparations of KSHV BAC plasmids were obtained from 500-ml *E. coli* cultures with the Qiagen Large construct kit (Qiagen, Inc., Valencia, CA) according to manufacturer instructions.

### Southern blot and junction PCR analysis

The DNA probe used for Southern blot hybridizations was amplified as a 1.4-kb fragment (corresponding to the RBP-Jκ locus of RTA promoter) with the KSHV genome as the template and primer set forward: 5′-ATGCAGCGGGGTGAGCCTGCCTCCAGCC-3′, and reverse, 5′-TTGCAGAATACTGGACAACAGCGCGTCG-3′. Purified KSHV BAC plasmid DNA was digested with XhoI and resolved on 0.65% agarose gels in 0.5X Tris-borate-EDTA buffer for 14 to 18 h at 40 V. DNA fragments were visualized by ethidium bromide staining, denatured, and transferred to Zeta-Probe GT genomic tested blotting membranes (Bio-Rad Inc, Hercules, CA). DNA probes were radiolabeled with [α-^32^P] dCTP with the NEBlot (New England Biolabs, Inc., Ipswich, MA). Prehybridization was performed at 63°C for 1 h in hybridization buffer (7% sodium dodecyl sulfate, 10% polyethylene glycol, 1.5X SSPE [1X SSPE is 0.18 M NaCl, 10 mM NaPO_4_, and 1 mM EDTA, pH 7.7]). DNA blots were hybridized with radiolabeled probes in the same solution at 63°C for about 7 h. Blots were washed twice for 15 min with 2X SSC (1x SSC is 0.15 M NaCl plus 0.015 M sodium citrate)-0.1% sodium dodecyl sulfate and twice for 30 min with 0.1X SSC-0.1% sodium dodecyl sulfate at 63°C. Blots were exposed to a Phosphoimager plate (Molecular Dynamics, Inc. Sunnyvale, CA) overnight at room temperature followed by scanning with the Typhoon 9200 (GE Healthcare Inc., Piscataway, NJ).

Junction PCRs were performed to identify the expected deletions. The primers used were RTA_1st_ (genomic position 69010 to 69291, NC_009333) 5′ TCCCAGCCAAGTCCCTCGTG 3′ and 5′ GTCCCACTGCTGCGATCCAG 3′; RTA_all_, (genomic position 69010 to 70537, NC_009333) 5′ TCCCAGCCAAGTCCCTCGTG 3′ and 5′ GCCCGGATACGCGCACATGC 3′.

### Reconstitution of recombinant viruses, virus induction and determination of virus copies

Purified Bac36 DNAs were transfected into 293 cells via CaPO4 method. Hygromycin B (150 ng/ml) was then added for selection 24 h after transfection. Three weeks after selection, homogenous populations of GFP-positive cells harboring KSHV Bac36 DNAs were obtained. Butyric Acid at a final concentration of 3 mM and TPA (Sigma) at 20 ng/ml was used for lytic induction. Cell suspensions were centrifuged at 3000 rpm for 20 min and the supernatant was filtered through a 0.45 µm cellulose acetate filter. The viral particles were concentrated by ultracentrifugation at 70,000xg at 4°C and stored at −80°C.

### Infection of PBMCs and TIVE cells with recombinant KSHV virions

Infection of PBMCs were performed as described previously [43]. In brief, 1x107 were infected by incubation with virus suspension in 1ml of RPMI 1640 (with 10% FBS) medium in the presence of Polybrene at a final concentration 5 ng/µl (Sigma, Marborough, MA) and incubated for 4 h in 37°C. Cells were centrifuged for 5min at 1500rpm, the supernatant discarded, pelleted cells were washed by fresh RPMI medium for 2 times and resuspended in fresh RPMI 1640 (10% FBS) medium in 6-well plates and culture at 5% CO2, 37°C humidified incubator.

TIVE cells were cultured in 12-well plates to 60% confluence in Medium 199 supplemented with 20% FBS, 200 mM L-glutamine, 5 mg/ml Penicillin/Streptomycin(P/S) and 10 mg/ml Endothelial cell growth supplement from bovine neural tissue. Concentrated viruses were added to the supernatant in the presence of Polybrene (4 µg/ml) and spun at 2,500 rpm for 1 h at room temperature. The supernatants were removed and washed twice and then incubated with fresh medium. GFP expression was used to monitor infection under fluorescence microscope (Olympus Inc., Melville, NY).

### Extraction and determination of intracellular and extracellular viral DNAs

For quantitation of intracellular viral DNA, cells were harvested and washed twice with 1xPBS to remove the residual viruses. Cells were incubated by HMW buffer (10mM Tris-HCl pH 8.0, 150mM NaCl, 10mM EDTA, 0.5% SDS) for 2 hrs at 55°C. 0.5 mg/ml proteinase K was added and incubated at 37°C overnight with subsequent extraction in phenol/chloroform/isopropanol. Viral DNA was treated with RNase, then precipitated and resuspended in water. Extracellular viral DNA was extracted from culture supernatants as essentially described previously [35], [43]. In brief, virions were pelleted down at 70,000xg for 2 hrs at 4°C and resuspended. Cellular DNAs and free viral DNAs were removed by treatment with DNase I at 37°C for 1∼2 hrs. Virion DNA was treated with HMW buffer for 20 minutes. Lysates were treated with proteinase K overnight at 37°C with subsequent extraction with phenol/chloroform/isopropanol. Intracellular and extracellular viral DNAs were quantitated by real-time DNA PCR for TR (5′ GGCTCCCCCAAACAGGCTCA 3′, and 5′ GGGGGACCCCGGGCAGCGAG 3′). GAPDH and BAC36 DNAs were the standards for intracellular and extracellular viral DNAs, respectively.

### Quantitation of KSHV RNA

Total RNA from infected PBMCs were extracted by using TRIzol (Invitrogen, Inc., Carlsbad, CA) and 1 µg DNase-treated total RNA were used to generate cDNA using the High capacity RNA-to-cDNA kit (Applied Biosystems Inc., Foster City, CA) according to manufacturer's instructions. RT-qPCR was performed on a StepOnePlus Real-Time PCR System (Applied Biosystems Inc, Carlsbad, CA) or Opticon 2 Real-Time PCR System. The reactions were carried out in a 96-well plate at 95°C for 10 min, followed by 35 cycles at 95°C for 30 s, 51°C for 30 s and then 72°C for 40 s. The differences of cycle threshold values (CT) between the samples (ΔCT) were calculated after standardization by GAPDH and converted to fold changes using one of the samples as a standard (1-fold). The primers used were LANA: 5′ CATACGAACTCCAGGTCTGTG 3′, 5′ GGTGGAAGAGCCCATAATCT 3′; RTA: 5′ CAGACGGTGTCAGTCAAGGC 3′, 5′ ACATGACGTCAGGAAAGAGC 3′; GAPDH 5′ GGTCTACATGGCAACTGT GA 3′, 5′ ACGACCACTTTGTCAAGCTC 3′. All the reactions were run in triplicates.

### Immunostaining

Cells were applied to a slide well and fixed with 4% paraformaldehyde with 0.1% Triton X-100, and blocked with 10% BSA. Cells were then incubated with a primary antibody (Mouse anti-LANA), and specific signals were detected with a secondary antibody conjugated with Alexa Fluor 594 (Invitrogen, Carlsbad, CA). The cells were counterstained with 4′, 6′-diamidino-2-phenylindole (DAPI). Images were observed and recorded with a Fluoview FV300 microscope (Olympus Inc., Melville, NY).

### Colony formation and growth assay

BAC36 wt, BAC RTA1st and BAC RTAall transfected 293 cells were selected for 3-4 weeks with Hygromycin B. 300 stably transformed 293 cells were seeded in 6 cm Petri dish in DMEM supplemented with 10% FBS, Hygromycin B (150 ng/ml) and Difco Noble Agar (BD, Franklin Lakes, NJ) (0.5%). After 2 weeks of growth, the colony were monitored and photographed under fluorescence microscope (Olympus Inc., Melville, NY). The plates were scanned by Typhoon 9200 for GFP signals and the colony number was quantitated using the Odyssey V3.0 software.

### Flow cytometry

BAC36 wt, BAC RTA_1st_ and BAC RTA_all_ stably transfected 293 cells were harvested and fixed in 1% paraformaldehyde for 30–60 min. The fixed cells were washed twice by 1XPBS and analyzed using on FACSCalibur based on GFP signals.

Infected PBMCs cells were stained essentially as described previously [46], [47]. Briefly, PBMCs were harvested and washed at 1dpi, 2dpi, 4dpi and 7dpi. T cells and B cells were detected by using the APC conjugated anti-CD3 and PercpCy 5.5 conjugated anti-CD19 mAbs (BD Biosciences, San Jose, CA). GFP signals were used to monitor KSHV positive cells. Data were acquired on FACSCalibur equipped with CellQuest Pro software and analyzed using FlowJo software.

### Statistics

Data are shown as mean values with standard errors of the means (SEM). The significance of differences in the mean values was evaluated by 2-tailed Student's t test. P<0.05 was considered statistically significant.

## Results

### Generation of recombinant KSHV viruses deleted for RBP-Jκ binding sites within the RTA promoter (RTA_1st_ and RTA_all_)

**Figure 1:**
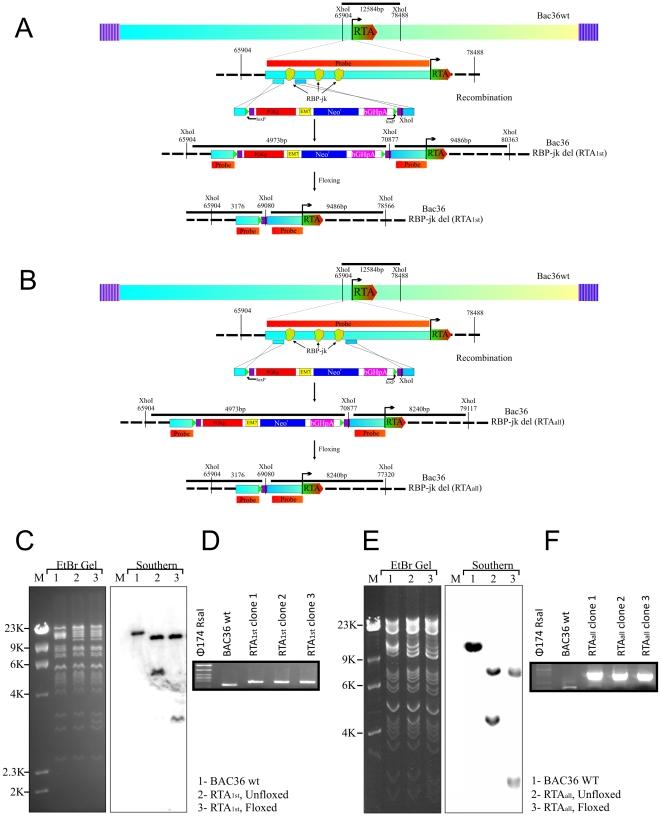
Generation of two recombinant KSHV BACmids with deletion of RBP-Jκ sites in the RTA promoter. (A) Schematic diagram showing generation of BAC RTA_1st_, a recombinant BAC36 with first RBP-Jκ site deletion in the RTA promoter (B) Schematic diagram showing generation of BAC RTA_all_, a recombinant BAC 36 with deletion of first, 2^nd^ and 3^rd^ RBP-Jκ site in the RTA promoter. (C) Ethidium bromide-stained gel and southern blots with BAC36 wt (lane 1) and the mutated BACmid, RTA_1st_ (Unfloxed, lane 2) and RTA_1st_ (floxed, lane 3), cleaved with XhoI. (D) PCR analysis for Bac36 wt and RTA_1st_ recombinant virus at the junction of deletion of RBP-Jκ site within RTA promoter. (E) Ethidium bromide-stained gel and southern blots with BAC36 wt (lane 1) and the mutated BACmid, RTA_all_ (Unfloxed, lane 2) and RTA_all_ (floxed, lane 3), cleaved with XhoI. (F) PCR analysis for BAC36 wt and RTA_1st_ recombinant virus at the junction of deletion of RBP-Jκ sites within RTA promoter. DNA fragments were sequenced and confirmed the mutation.

Our previous studies showed that RTA contributes to establishment of KSHV latency by activating LANA expression during the early stages of infection via RBP-Jκ, the major effector of the Notch signaling pathway [28]. The activity of the RTA promoter was reduced about 40% in the truncations of first RBP-Jκ and all three RBP-Jκ binding site within RTA promoter compared to the wt promoter [30]. To investigate the roles of the RBP-Jκ binding sites in the RTA promoter, we constructed two KSHV recombinant viruses, BAC RTA_1st_ with a deletion of the first RBP-Jκ binding site and RTA_all_ with deletion of all three RBP-Jκ binding sites. BAC36 wt carries the full KSHV genome, a GFP tag, and a eukaryotic resistance gene, hygromycin [48]. Infectious KSHV can be reconstituted by transfection of BAC36 wt DNA into 293 cells [48]. Using the BAC36 wt as a template, we designed PCR primers so that the RBP-Jκ binding sites in the RTA promoter were removed from the genome. Fig. 1A and B shows a schematic for the generation of the recombinant BAC RTA_1st_ and RTA_all_ using PCR primers that integrated the Kanamycin resistance gene (Neo: the neo cassette is resistant to Neomycin or Kanamycin in prokaryotes) and two loxP sites from plasmid pL452 into the BAC36 genome, replacing the RBP-Jκ binding sites of the RTA promoter. LoxP is the substrate sequence of Cre recombinase, so the insert fragment between two loxP sites (including neo cassette) can be subsequently removed by expressing Cre recombinase after induction by L-arabinose [49]. A PCR product containing Neo^r^ flanked by loxP sites and two fragments of 50-bp KSHV sequences from the two ends of the RBP-Jκ site in the RTA promoter was generated using pL452 plasmid as a template. This PCR product was transfected into BAC36 wt-E.coli 350 to remove the RBP-Jκ site in the RTA promoter after homologous recombination and Cre-mediated excision of Neo^r^. The resulting BAC recombinants were screened and analyzed on 0.65% agarose and subsequently by southern blot analysis to show that the RBP-Jκ binding site in the RTA promoter was removed from the KSHV genome (Fig. 1A and C). Digestion of the BAC36 wt DNA with XhoI generated one 12584kb fragment at the RBP-Jκ binding site in the RTA promoter. For BAC RTA_1st,_ replacement of the RBP-Jκ binding site with the Kan cassette changes the two fragment sizes to 9486bp and 4973bp. After induction, the fragment between two loxP sites was removed, so the smaller fragment (4973b) shifted in size to 3176kb, indicating removal of Kan cassette. Southern blot showed the presence of a 5kb band before induction and a unique 3kb band in recombinant BAC RTA_1st_ when hybridized with a probe within the RBP-Jκ binding site (Fig. 1A and C). To further confirm whether the altered digestion pattern of the BAC mutants was the result of the expected recombination, we carried out junction PCR by using the primers designed at the recombination site showing that the junction bands in the BAC RTA_1st_ shifted based on the presence of the remaining loxP site and XhoI site (Fig. 1D). Similarly, For BAC RTA_all,_ replacement of the RBP-Jκ binding sites with the Kanamycin cassette changes the two fragment sizes to 8240bp and 4973bp. After induction, the fragment between two loxP sites was removed, so the smaller fragment (4972b) shifted in size to 3176kb - indicating removal of the Kan cassette. Southern blot showed the presence of a 5kb band before induction and a unique 3kb band in the recombinant BAC RTA_all_ when hybridized with a probe within the RBP-Jκ binding site (Fig. 1B and E). Junction PCR showed that the junction bands in the BAC RTA_all_ shifted based on the presence of the remaining loxP site and XhoI site (Fig. 1F). Finally, the PCR products were sequenced to confirm the expected mutation.

### BAC RTA_1st_ and RTA_all_ recombinant KSHV stable 293 cells had a decreased capability for lytic replication

**Figure 2:**
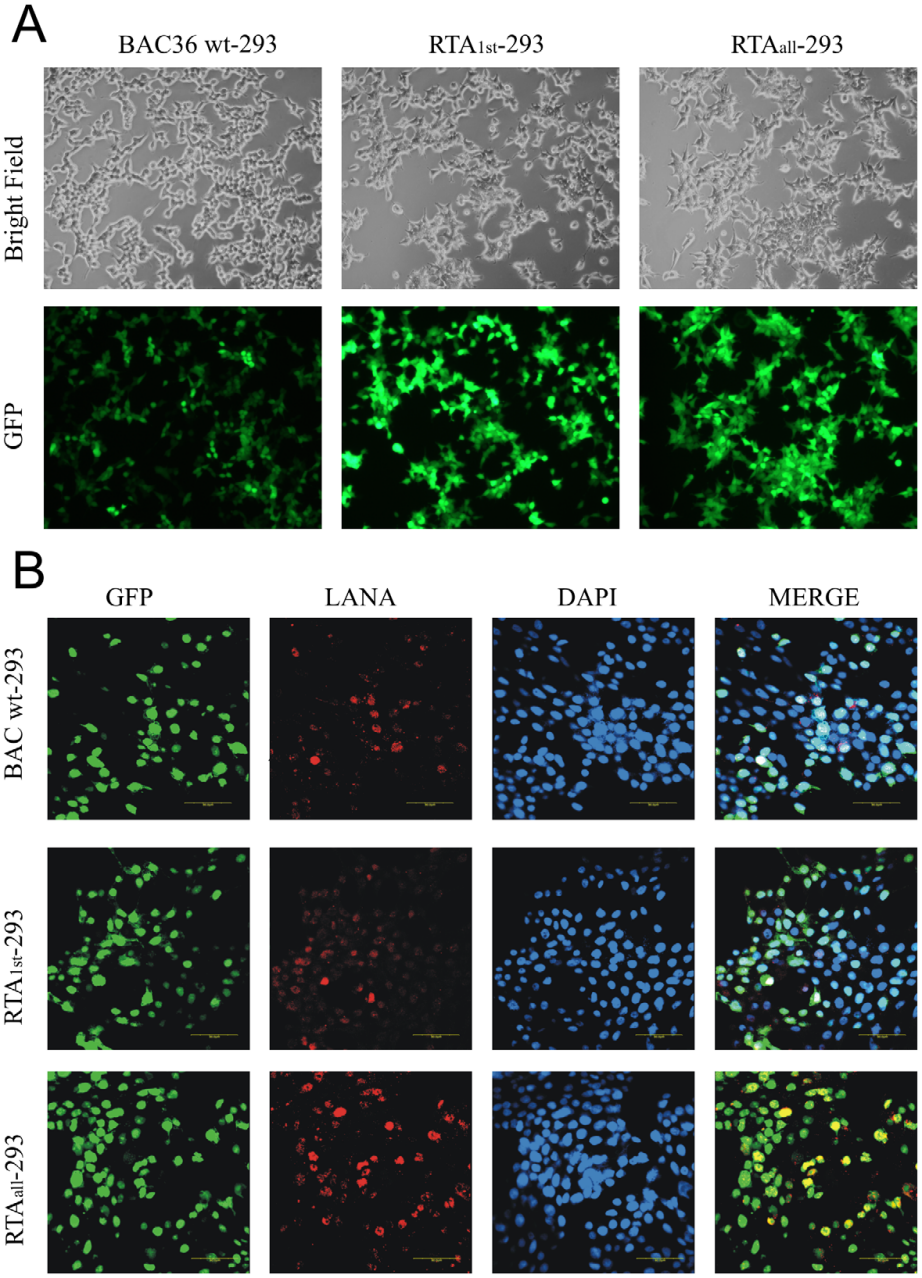
Transfection of 293 cells with BAC36 wt, BAC RTA_1st_ and BAC RTA_all_ DNAs. (A) Cells were transfected with BAC36 wt, BAC RTA_1st_ and BAC RTA_all_ DNAs. GFP expression levels were monitored by fluorescent microscopy 2 days after transfection, and the transfected cells were split and selected with Hygromycin B. The hygromycin-resistant cells were pooled and passed three to four times. The homogenous population of GFP-positive cells harboring KSHV wild-type and mutant genomes were obtained. (B). Immunofluorescence analysis for LANA in BAC36-293 BAC RTA_1st_-293 and BAC RTA_all_-293 cells.

To reconstitute recombinant viruses, we transfected BAC36 wt, RTA_1st_ and RTA_all_ DNAs into 293 cells. The transfection efficiencies were monitored by the fluorescence microscopy. GFP positive cells were detected after 24–48h post-transfection (data not shown). Positive cells were enriched by hygromycin selection, generating 293 cell lines bearing BAC36 wt, RTA_1st_ and RTA_all_ (Fig. 2A). Subsequently, the cells were fixed and immunostained against LANA to confirm that BAC 36 wt and RTA_1st_ and RTA_all_ stable cell lines harbored the KSHV genome (Fig. 2B). Similar levels of GFP positive signals and LANA staining were observed for all three stably transfected cell lines generated due to hygromycin selection.

**Figure 3:**
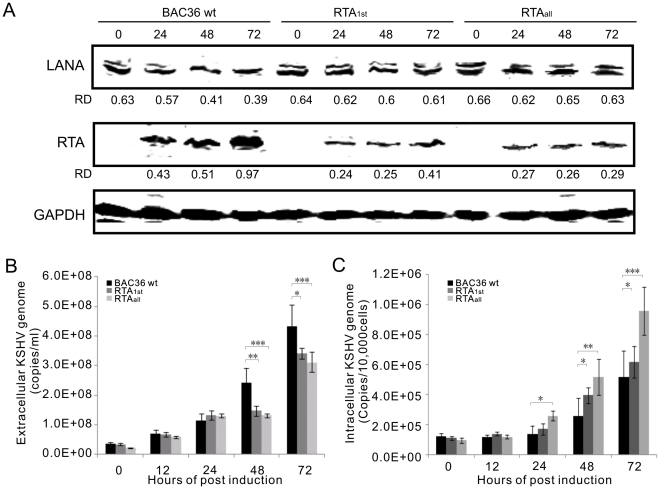
Comparisons of effect on deletion of RBP-Jκ sites within RTA promoter in KSHV lytic life cycle. (A) Comparison of levels of viral gene expression of BAC36 wt, RTA_1st_ and RTA_all_ recombinant viruses in 293 cells. Western blot for RTA and LANA at 0, 24, 48, 72 hours lytic induction of BAC36 wt, RTA_1st_ and RTA_all_ stable 293 cell lines in the presence of Butyrate acid and TPA. The same blot was also reprobed with anti-GAPDH antibody to ensure equal loading of each sample. RD: relative density. (B) Detection of intracellular KSHV viral genome copies using TR primer in BAC36 wt-293, RTA_1st_-293 and RTA_all_-293 cells induced by Butyrate acid and TPA. (C) Detection of extracellular KSHV progeny virion production using TR primer in the supernatant of BAC36 wt-293, RTA_1st_-293 and RTA_all_-293 induced by Butyrate acid and TPA. *P<0.05; **P<0.01; ***P<0.001 by Student's t test.

To ensure that both RTA_1st_ and RTA_all_ stable cells were able to produce recombinant viruses after lytic reactivation, the cells were treated with TPA and butyric acid to induce lytic reactivation. Whole cell lysates were prepared from the uninduced and induced cells at 24, 48, 72 hours post induction (hpi), and the expression of LANA and RTA were analyzed by western blot analysis using the corresponding specific antibodies. The results showed that LANA expression exhibits a slight decrease after induction in BAC36 wt-293 cells at 48 and 72 hpi as more cells switch to lytic replication. However, there was no obvious difference in RTA1st and RTAall-293 cells (Fig. 3A). This may be due to the fact that more cells were infected with RBP-Jκ mutant viruses in latent phase with higher levels of LANA. In the wild type, RTA expression was not affected thus most of the viral genome copies underwent lytic reactivation thereby expression of latent protein, LANA diminished over time. This is further evident by the levels of RTA, which increased, in a time dependent manner after induction with TPA and butyric acid, in wt BAC36-293 cells (Fig. 3A). Notably, though RTA expression was increased in a time-dependent manner after induction in all cell lines, the levels of RTA expression in RTA_1st_ and RTA_all_-293 was much lower than seen in BAC36 wt-293 cells. This suggests that RTA expression was reduced in RTA_1st_ and RTA_all_-293 cells (Fig. 3A). In addition, the viruses were collected from supernatant of the induced BAC36 wt, RTA_1st_ and RTA_all_-293 cells and quantitated for virion particles by quantitative PCR analysis. The results showed that RTA_1st_ and RTA_all_-293 cells produced fewer KSHV genomes, suggesting a decrease in virion production post-induction (Fig. 3B). This confirms our hypothesis that deletion of the RBP-Jκ binding sites in the RTA promoter of KSHV results in an attenuated lytic cycle and thus decreased viral progeny. Furthermore, total DNA was extracted from BAC36 wt, RTA_1st_ and RTA_all_-293 cells. Intracellular viral DNA levels were determined by quantitative PCR analysis standardized by GAPDH. The result showed that RTA_1st_ and RTA_all_-293 cells had a greater number of viral copies compared to BAC36 wt-293 cells, further indicating that RTA_1st_ and RTA_all_ deficient viruses exhibited a decrease in lytic capability although the genome copy numbers were greater (Fig. 3C).

### RTA_1st_ and RTA_all_ recombinant KSHV showed enhanced latency and promoted cell growth and proliferation of 293 cells

**Figure 4:**
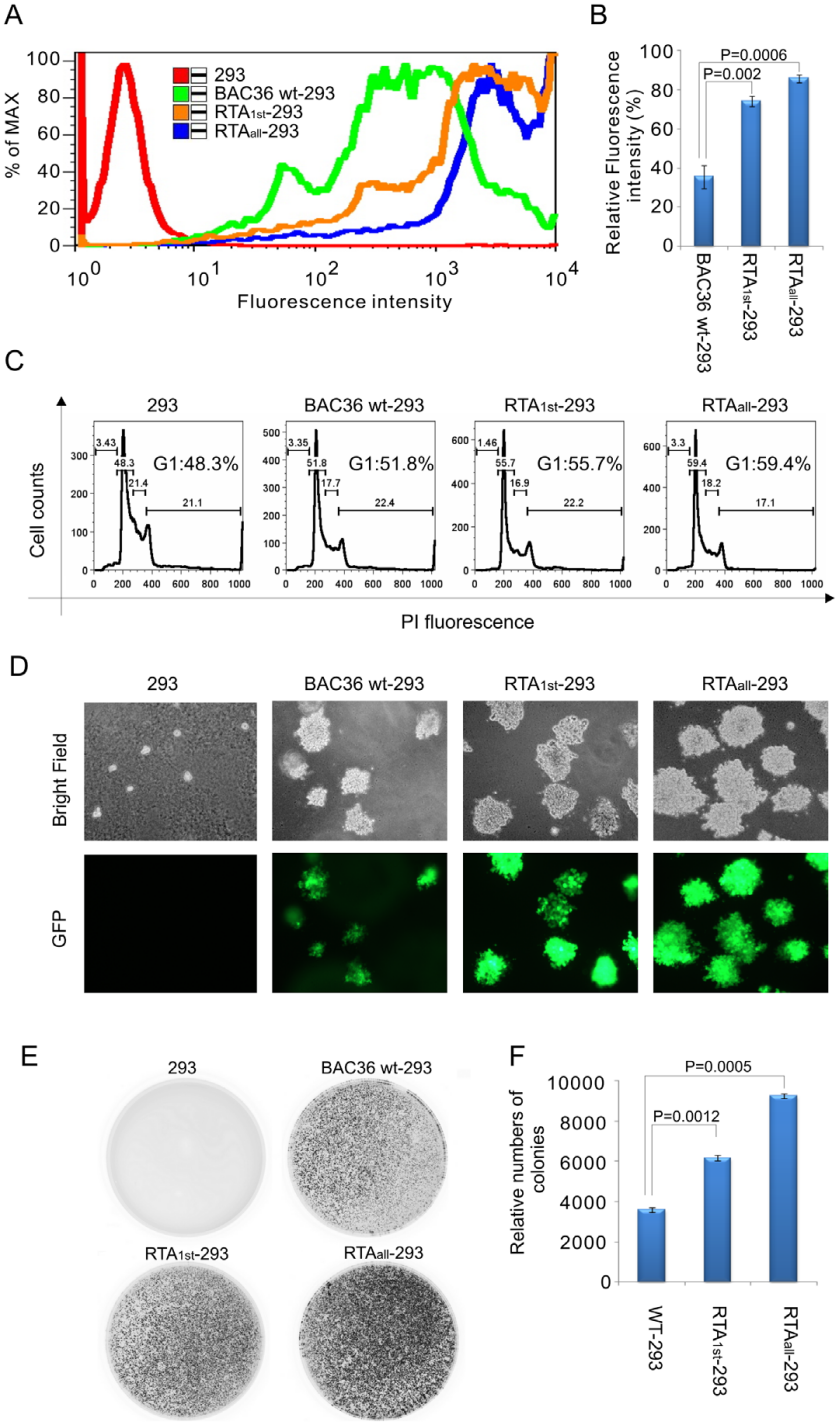
Comparison of cell growth and proliferation for WT, RTA_1st_ and RTA_all_ -293 cells. (A) BAC36 wt, RTA_1st_ and RTA_all_ transfected 293 cells selected for 3–4 weeks were assayed by FACS analysis based on GFP signals. (B) Relative GFP density in the BAC36 wt, BAC RTA_1st_ and BAC RTA_all_ transformed 293 cells. (C) BAC36 wt, RTA_1st_ and RTA_all_-293 cells were starved in DMEM with 0.1% FBS for overnight. Next day, media were replaced with DMEM supplement with 5% FBS. Cells were cultured for 24 hrs and harvested for analysis by flow cytometer. (D) 293 cells transfected with BAC36 wt, BAC RTA_1st_ and BAC RTA_all_ viruses were selected with Hygromycin for a 4-week selection, cells were seed on the plates. Colony were monitored and photographed under fluorescence microscopy after growth for two weeks. (E) The plates were scanned by Typhoon 9200 based on GFP signals. (F) The colony number was quantized using odyssey V 3.0.

BAC36 wt, RTA_1st_ and RTA_all_ are recombinant KSHV viruses harboring a GFP marker which allows us to track viral genome stability in 293 cells. Furthermore, RTA_1st_ and RTA_all_ 293 cells possessed higher intracellular viral DNA copies than BAC36 wt-293 cells. We postulated that both of them should have increased GFP signals. Flow cytometry was performed based on GFP signals for BAC36 wt, RTA_1st_ and RTA_all_ -293 cells. Interestingly, RTA_1st_ and RTA_all_-293 cells showed approximately 78% and 93% GFP fluorescence intensity, respectively. However, only 41% GFP fluorescence intensity was seen for BAC36 wt-293 cells (Fig 4A, B) and pellets from collected RTA_1st_ and RTA_all_-293 cells also had a more intense green color based on visual inspection when compared to BAC36 wt-293 cells (data not shown).

KSHV is a known human oncovirus and is associated with cellular transformation [31], [50], [51]. Therefore we wanted to determine the proliferation rate for BAC36 wt, RTA_1st_ and RTA_all_-293 cells. Cells were starved in DMEM with 0.1% FBS overnight. Next day, media were replaced with DMEM supplemented with 5% FBS. Cells were cultured for 24 hrs and harvested for analysis by flow cytometer. Interestingly, RTA_1st_ and RTA_all_-293 cells had a higher percentage of cells (55.7% and 59.4%) comparing to BAC36 wt-293 (51.8%) in G1 phase which was consistant after multiple repeats (Fig. 4C). These results suggested that RTA_1st_ and RTA_all_ recombinant viruses can promote cell growth and proliferation in the infected cells. In addition, cells harboring more viral genome copies should have an enhanced capability for driving cell growth. Therefore we tested this hypothesis with a colony formation assay. The colonies were photographed using fluorescence microscopy and scanned by a Typhoon 9200 system based on GFP signals. Surprisingly, the average size of hygromycin-resistant colonies for RTA_1st_ and RTA_all_-293 cells was distinctively bigger (almost double) relative to BAC36 wt-293 cells (Fig. 4D), indicating that RTA_1st_ and RTA_all_ recombinant viruses possess enhanced capability for cell growth. Furthermore, GFP positive colonies were scanned and quantitated. The results also showed that RTA_1st_ and RTA_all_-293 cells showed a 1.8 and 2.4 fold increase in total colonies in comparison to BAC36 wt-293 (Fig. 4E, F), providing further evidence that mutation of the RBP-Jκ sites enhanced KSHV latent infection and so promoted cell growth and proliferation.

### RTA_1st_ and RTA_all_ recombinant viruses have enhanced latent infection in human peripheral blood mononuclear cells (PBMCs)

**Figure 5:**
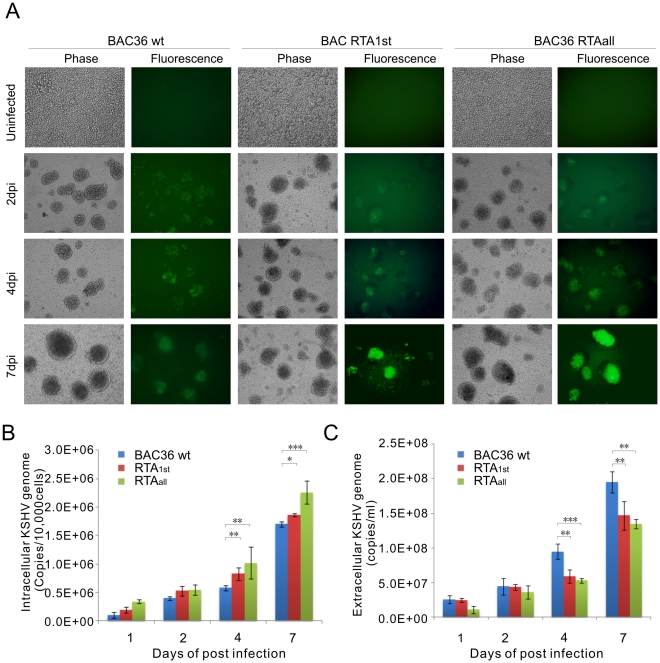
Comparisons of relative infectivity for BAC36 wt, RTA_1st_ and RTA_all_ recombinant viruses. (A) PBMCs were infected by KSHV BAC36 wt, RTA_1st_ and RTA_all_ viruses with equally loading. GFP expression was monitored under a fluorescent microscope after 2dpi, 4dpi and 7dpi. (B) Intracellular KSHV viral genome copies were measured by a real-time PCR with primers to TR at 1 dpi, 2 dpi, 4 dpi and 7 dpi. (C) Extracellular KSHV progeny virion progenies were analyzed by a real-time PCR with primers to TR at 1 dpi, 2 dpi, 4 dpi and 7 dpi. Dpi, days post infection. *P<0.05; **P<0.01; ***P<0.001 by Student's t test.

KSHV infection is strongly linked to two lymphoproliferative diseases, referred to as PEL and MCD [1], [2]. Infection of PBMCs with KSHV can provide greater insights into initiation and development of KSHV associated lymphomagenesis. KSHV can infect many cell types, including epithelial cells, keratinocytes, endothelial cells and PBMCs which are permissive cells with varying degrees of infectivity [36], [44], [52]. Previous studies show that determination of GFP signals can be effectively used to monitor the efficiency by which KSHV BAC36 infects PBMCs [43]. Our investigation shows that mutations of the RTA promoter, specifically the RBP-Jκ cognate sequences, results in reduced RTA expression as well as reduced RTA-mediated auto-activation of its promoter [30]. Here, we have now developed a BAC system to further explore the feedback regulation of RTA by LANA. As described previously, GFP signals were monitored by green fluorescence from the infected PBMCs [43]. We concentrated BAC36 wt, RTA_1st_ and RTA_all_ viruses from TPA and Butyric acid treated-293 cells. The concentrated viruses were used to infect PMBCs and the infected cells were monitored at 1, 2, 4 and 7 days post infection (dpi). The expected result was that virion particles from both BAC36 wt and recombinant viruses would be detected as GFP signals were seen by 2dpi (Fig. 5A). Infected PBMCs were collected at 1, 2, 4 and 7dpi and total DNA was extracted as indicated in materials and methods. Viral DNA levels were determined by real-time PCR normalized to endogenous GAPDH. As indicated by GFP signals, viral copies of BAC36 wt showed a time-dependent manner increase in viral DNA, and the viral DNA copies of RTA_1st_ and RTA_all_ recombinant viruses had a similar level at 1 and 2 dpi (Fig. 5B). However, genome copies of RTA_1st_ and RTA_all_ recombinant viruses showed a significant increase in infected PBMCs above that of BAC36 wt at 1dpi, 2dpi, 4dpi and 7dpi, suggesting that more latent viral genomes were tethered to the host genome (Fig. 5B). Extracellular viral DNAs were collected from infected supernatants and quantitated by using KSHV TR primer set. In contrast, viral DNA levels of RTA_1st_ and RTA_all_ recombinant viruses were decreased in comparison to BAC36 wt from 1dpi to 7dpi, indicating that the lytic cycle of the recombinant viruses was not as robust. These data further support our pervious results showing that RTA_1st_ and RTA_all_ recombinant viruses exhibited an enhanced latent infection phenotype and a reduced capability for lytic replication in this system (Fig 5C).

### RTA_1st_ and RTA_all_ recombinant viruses have reduced capability for inducing lytic replication after primary infection

**Figure 6:**
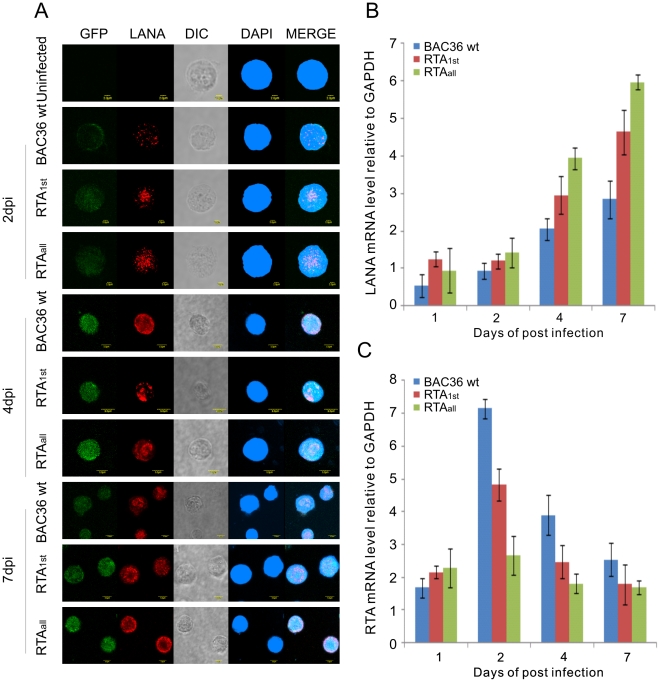
Quantitative analysis of KSHV BAC36 wt, RTA_1st_ and BAC RTA_all_ recombinant viruses infected PBMCs at 1dpi, 2dpi, 4dpi and 7dpi. (A) Immunofluorescence assay for PBMCs infected by KSHV BAC36 wt, RTA_1st_ and RTA_all_ recombinant viruses at 2 dpi, 4 dpi and 7 dpi. Uninfected and infected PBMCs at 2 dpi, 4 dpi and 7 dpi were stained for LANA protein expression. PBMCs expressed GFP, indicating the presence of viral genome. (B, C). Quantitative analysis for determination of latent and lytic infection in PMBC cells infected by KSHV BAC36 wt, RTA_1st_ and RTA_all_ recombinant viruses. Total RNAs were extracted, treated with DNase I, and reverse transcribed to cDNA after 1 dpi, 2 dpi, 4 dpi and 7dpi. Quantitative Real-time PCR analysis with the primers for LANA and RTA was performed using StepOnePlus Real-Time PCR System. Error bars indicate standard deviations from three separate experiments.

**Figure 7:**
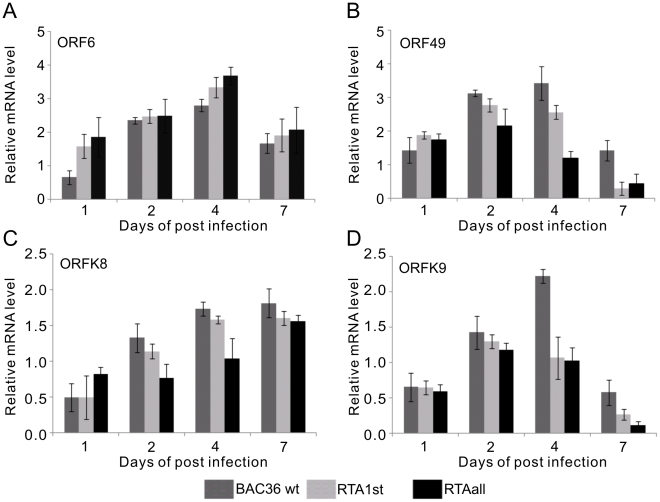
Quantitative analysis of ORF 49, ORF6, K8 and K9 expression in KSHV infected PBMCs at 1dpi, 2dpi, 4dpi and 7dpi. Total RNAs were prepared and transcribed to cDNA and the qRT-PCR analysis with the primers for (A) ORF 49, (B) ORF6, (C) K8 and (D) K9 was performed using the Power SYBR green PCR master mix with GAPDH as control. Error bars indicate standard deviations from three separate experiments.

LANA and RTA are viral encoded molecular switches for latent and lytic phases in KSHV infection. We wanted to determine the mRNA levels of RTA and LANA during primary infection. Immunofluorescence assays showed that LANA signals were detected in BAC36 wt, RTA_1st_ and RTA_all_ infected PBMCs at 1 dpi, 2 dpi, 4 dpi and 7dpi indicating a successful infection (Fig. 6A). DNase treated total RNA from infected PBMCs at 1 dpi, 2 dpi, 4 dpi and 7dpi were used to generate cDNA and signals of RTA and LANA transcripts quantitated by real-time PCR. The results showed that LANA expression from BAC36 wt infected PBMCs increased in a time-dependent manner. Furthermore, RTA_1st_ and RTA_all_ recombinant viruses infected PBMCs showed higher LANA mRNA expression from 1 dpi to 7dpi, indicating that deletion of the RBP-Jκ cognate sequences within the RTA promoter can result in greater stringency for latent infection (Fig. 6B). Additionally, mRNA of RTA was also analyzed and showed a peak at 2dpi in BAC36 wt and recombinant viruses (Fig. 6C), perhaps promoting lytic infection at an early stage [47]. However, compared to BAC36 wt, RT-PCR results from RTA mRNA showed lower levels in RTA_1st_ and RTA_all_ infected PBMCs from 1 dpi to 7dpi with almost no significant change with RTA_all_ recombinant virus (Fig. 6C). This suggests that deletion of the RBP-Jκ cognate sequences in the promoter led to a dramatic loss in the ability of the recombinant viruses to induce lytic cycle activation, and as a result more tightly maintain latent infection (Fig 6C). Furthermore, KSHV ORF6 which encodes the single-stranded DNA (ssDNA) binding protein and is tightly associated with DNA replication, showed increased expression in RTA_1st_ and RTA_all_ infected PBMCs compared to BAC36 wt infected PBMCs (Fig 7A). This suggests that KSHV replication is actively engaged although latent infection is more stringent (Fig 7A). ORF49 encoded by KSHV lies adjacent to and is transcribed in the opposite orientation to RTA. It also co-operates with RTA to activate KSHV lytic cycle [53]. We also monitored the activity of ORF49, which cooperates with RTA during lytic replication through activation of several lytic promoters containing AP-1 sites [53]. The mRNA level was initially higher for recombinant viruses at 1dpi (Fig 7B). However, at 4dpi and 7dpi, the ORF49 transcript levels for the recombinants were much lower than BAC36 wt with RTA_all_ greater than 2-fold less at 4dpi. By 7dpi, both RTA_1st_ and RTA_all_ were further depressed compared to BAC36 wt to about 3-fold (Fig. 7B). This suggests that active lytic replication of KSHV was dramatically reduced by 7dpi with a greater propensity for maintaining latency. We then investigated changes in the K8 and K9 transcript levels. The early lytic protein K8 [54], showed a general increase in transcript levels over the 7 day period. However, the levels of K8 transcripts for RTA_1st_ and RTA_all_ were generally lower than that of the BAC36 wt virus (Fig 7C). Interestingly, ORF K9 which encodes for vIRF, a homolog to members of the interferon (IFN) regulatory factor (IRF) and important for regulating intracellular interferon signal transduction [55], increased dramatically by 2 days, with BAC36 wt continuing to increase in K9 transcript levels. At 7 days all viruses showed a drastic reduction in K9 transcript levels (Fig. 7D). These results suggest a level of regulation of these transcripts which is important for controlling latent infection in KSHV.

### RTA_1st_ and RTA_all_ recombinant viruses showed enhanced ability to infect T and B cells during primary infection

**Figure 8:**
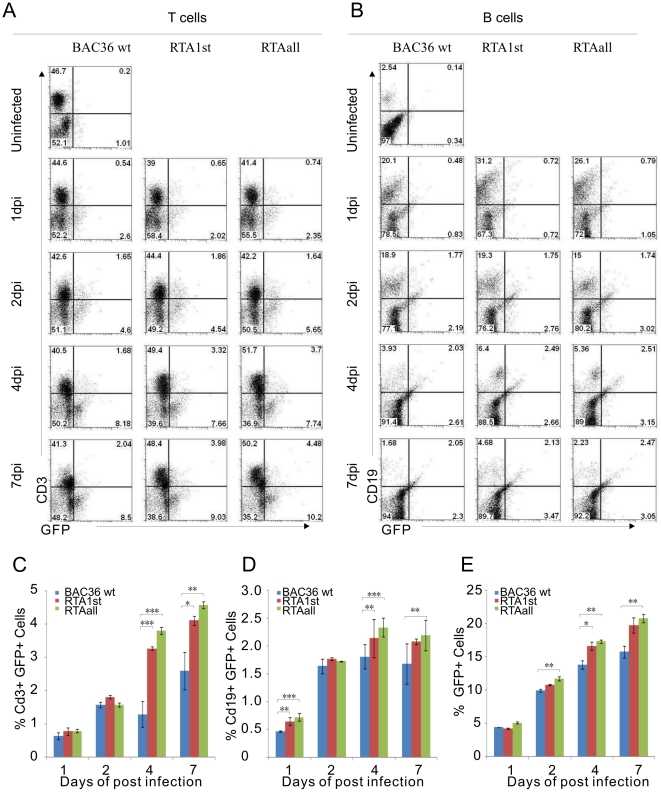
FACS analysis of T cells, B cells and GFP-positive cells in BAC36 wt, RTA_1st_ and RTA_all_ recombinant viruses infected PBMCs. (A, B) KSHV BAC36 wt, RTA_1st_ and RTA_all_ viruses infected PBMCs were harvested at 1dpi, 2dpi, 4dpi and 7dpi. T cells and B cells were detected by using the APC–conjugated anti-CD3 and PercpCy 5.5 -conjugated anti-CD19 mAbs. GFP signals were monitored KSHV positive cells. Data were acquired on FACSCalibur equipped with CellQuest Pro software and analyzed using FlowJo software. (C) KSHV infected T cells (CD3+ GFP+). (D) KSHV infected B cells (CD19+ GFP+). (E) Total KSHV infected PBMC cells (GFP +). *P<0.05; **P<0.01; ***P<0.001 by Student's t test (N = 9).

Human PBMCs contains lymphoid cells consisting of both T and B cells. The results above showed that RTA_1st_ and RTA_all_ recombinant viruses have an enhanced propensity for latent infection during the early stages of in vitro infection. Recently, Myoung and Ganem showed that 20–40% T and 4%–5% B cells from human tonsillar cultures can be infected [36]. However, only B cells support viral replication and produce progeny. Here, we are interested in the ability of KSHV to infect T and B cells from PBMCs during early infection. APC–conjugated anti-CD3 and PercpCy 5.5-conjugated anti-CD19 mAbs were used to detect infected T and B cells, respectively. GFP signals were used to detect KSHV-positive cells. Our results showed that GFP positive T cells were detected as early as 1 dpi (Fig 8A). The GFP positive T cells infected by BAC36 wt virus was slightly changed relative to RTA_1st_ and RTA_all_ recombinant viruses. At 2 dpi, the proportion of GFP+ CD3+ T cells was 1.65%, 1.86% and 1.64%, respectively. The percentage of GFP+ CD3+ T cells infected by BAC36 wt, RTA_1st_ and RTA_all_ recombinant viruses were increased to 1.68%, 3.32% and 3.7% at 4 dpi, respectively. At 7 dpi, T cells were infected continuously in a time-dependent manner and the proportion of GFP+ CD3+ T cells were 3.98% and 4.48% for RTA_1st_ and RTA_all_ relative to 2.04% for BAC36 wt (Fig 8A). These results further confirms that T cells were infected and that mutation of RBP-Jκ sites within RTA promoter can result in an increase in T cell infection as determined by GFP signals.

We then investigated the response of B cells exposed to KSHV. At 1 dpi the percentage of GFP+ CD19+ B cells were 0.48% for BAC36 wt, 0.72% for RTA_1st_ and 0.79% RTA_all_ recombinant viruses (Fig 8B). The next day, the proportion of GFP+ CD19+ B cells increased to 1.77% for BAC36 wt, 1.75% for RTA_1st_ and 1.74 % RTA_all_. At 4 dpi, the percentage of GFP+ CD19+ B cells further increased to 2.03% for BAC36 wt, 2.49% for RTA_1st_ and 2.51% RTA_all_, and at 7dpi, the GFP+ CD19+ B was similar to that at 4dpi suggesting that little or no further increase in B cell infection was seen. Furthermore, RTA_1st_ and RTA_all_ recombinant viruses infected B cells (2.13% and 2.47%) show a consistently higher rate of infection compared to BAC36 wt infected B cells (2.05%) (Fig 8B). This suggests that PBMCs were continually infected over the 7day period. Interestingly, GFP+ CD3+ T cells had a higher rate of infection compared to GFP+ CD19+ B cells from the BAC36 wt infected PBMCs (Fig 8C). Importantly, RTA_1st_ and RTA_all_ recombinant viruses possessed a higher infectivity compared to BAC36 wt virus for both infected T and B cells (Fig 8D). The overall amount of the GFP positive cells showed a definite increase in a time-dependent manner (Fig 8E). This further supports our previous data showing increased fluorescent signal from 2dpi to 7dpi. In general when compared to BAC36 wt, RTA_1st_ and RTA_all_ recombinant viruses infected PBMCs showed more GFP-positive cells, suggesting an increased ability for infection and maintenance of the KSHV genome within the first 7days after infection (Fig 8E).

### RTA_1st_ and RTA_all_ recombinant viruses showed an increased capability for infection and proliferation in long-term-infected telomerase-immortalized endothelial cells

**Figure 9:**
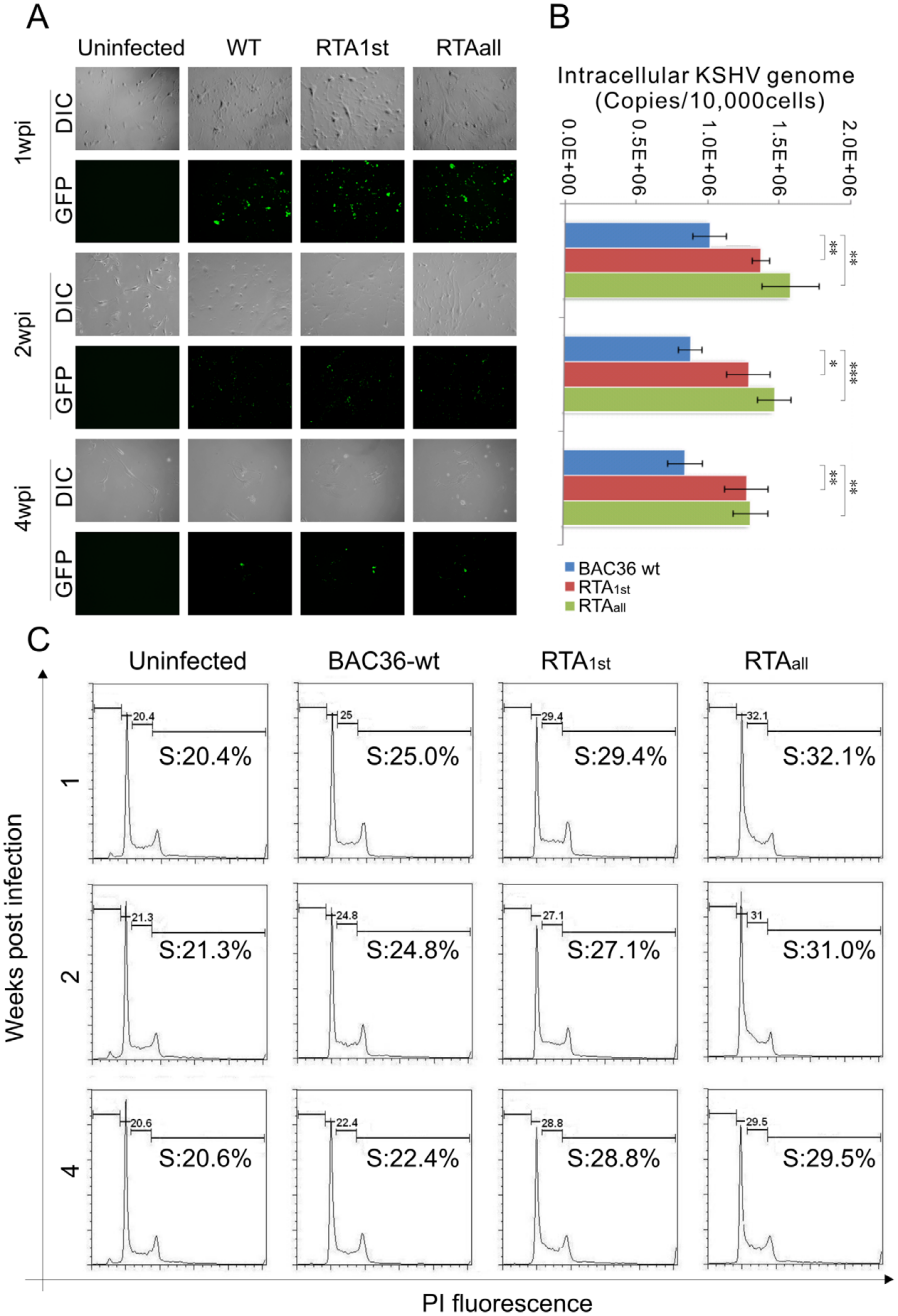
Analysis of KSHV BAC36 wt, RTA_1st_ and RTA_all_ recombinant viruses infected TIVE cells at 1, 2 and 4 weeks post infection (wpi). (A) TIVE cells were infected with concentrated KSHV BAC36 wt, RTA_1st_ and RTA_all_ recombinant viruses as described in materials and methods. GFP expression was used to monitor infection under fluorescence microscope at 1, 2 and 4 wpi. (B) Intracellular KSHV viral genome copies were measured by a real-time PCR with primers to TR at 1, 2 and 4 wpi. (C) Flow cytometer was performed for KSHV wt, RTA_1st_ and RTA_all_ recombinant viruses infected TIVE cells at 1, 2 and 4wpi. Wpi, week post-infection. *P<0.05; **P<0.01; ***P<0.001 by Student's t test.

Typically, infected PBMCs stopped clumping and most cells begin to die after 7 dpi. In our experiments, no transformation and/or immortalization was observed in infected PBMCs in vitro during the 7-day period. Thus it was difficult to monitor the effect of long-term infection of RTA_1st_ and RTA_all_ recombinant viruses. Here, we used recently developed telomerase-immortalized endothelial cells (TIVE) to monitor the ability of RTA_1st_ and RTA_all_ recombinant viruses to infect TIVE cells [44]. TIVE cells were infected with BAC36 wt, RTA_1st_ and RTA_all_ recombinant viruses as indicated in the materials and methods. GFP signals confirmed latent infection by BAC36 wt, RTA_1st_ and RTA_all_ viruses and photographs were taken at 1 week post-infection (wpi), 2wpi and 4wpi (Fig. 9A). We determined the copy number of KSHV genomes in infected TIVE as a measure of the persistence of the genomes. Total DNAs were extracted from BAC36 wt, RTA_1st_ and RTA_all_ recombinant viruses infected TIVE cells at 1, 2, 4wpi. Intracellular viral DNAs were determined by a quantitative PCR analysis standardized by GAPDH. The result showed that copy numbers of BAC36 wt, RTA_1st_ and RTA_all_ recombinant viruses were a slightly lower at 4wpi compared to 1 and 2wpi suggesting a possible loss of KSHV genome due to genome tethering instability. This phenomenon was also seen in BCBL-1-derived cell-free virus infected TIVE cells [44]. However, RTA_1st_ and RTA_all_ recombinant viruses maintained a similar copy number in the infected TIVE cells and infection levels were consistently greater than the BAC36 wt infected TIVE cells at 1 to 4wpi (Fig. 9B). These results further indicated that RTA_1st_ and RTA_all_ recombinant viruses exhibited a decrease in lytic capability. Previous studies showed that KSHV long-term-infected telomerase-immortalized endothelial cells exhibit a significant increase in number of cells in the S phase [44]. Here, we cultured infected TIVE cells for 4 weeks, harvested, fixed and stained them with propidium iodide. Flow cytometry analysis showed that the RTA_1st_ and RTA_all_ infected TIVE cells had an increased S phase population (29.4% and 32.1%), relative to BAC36 wt (25%) at 1wpi. Therefore, the recombinant viruses infected cells showed an enhanced capability to proliferate (Fig 9C). Similar patterns were seen at 2wpi and 4wpi where RTA_1st_ and RTA_all_ all exhibited increased S phase populations, further supporting our previous hypothesis that RTA_1st_ and RTA_all_ recombinant viruses can increase cell proliferation in TIVE cells.

## Discussion

KSHV RTA is an immediate early protein (IE) that initiates KSHV lytic reactivation from latent infection. It can directly or indirectly stimulate the transcription of a cluster of lytic genes as a transcription factor through binding to specific promoter sequences. RTA is a key regulator for KSHV reactivation because its expression is sufficient to activate the entire lytic cycle. Therefore understanding the regulation of RTA is to provide a better clue related to KSHV infection and tumorigenicity. RTA-deficient viruses are able to establish latency but are unable to reactivate [23]. Here, in an effort to explore KSHV latency and reactivation we generated two recombinant viruses which possess different latency and reactivation profiles compared to BAC36 wt. They serve as important reagents which allow us to examine the early-stages of KSHV infection. This provides a model with which to understand the development of KSHV-associated lymphoproliferative diseases.

RTA is an IE protein and its expression is also affected by other viral or cellular factors. For example, RTA up-regulates its own expression through interaction with the CCAAT/enhancer binding protein alpha(C/EBPα) at its promoter [20], [56]. Furthermore, RTA can down-regulate its ability for activating specific viral promoters by cooperating with the viral protein b-Zip, an early protein encoded by ORF K8 [57], [58]. Our previous studies showed that loss of one of those sites can potentially affect the overall regulation of all four RBP-Jκ sites within the RTA promoter [30]. Importantly, the observation that RTA_1st_ and RTA_all_ recombinant viruses enhance latency and can promote cell growth in 293 cells caused us to further investigate these mutations during early infection.

Many human cancers are associated with tumor viruses [59] and many are detected in PBMCs. Human papillomavirus (HPV) DNA has been found as an episomal form in PBMCs, but no transcripts are detected [60], [61]. Recent studies showed that PBMCs of haematuric cattle are additional reservoir of bovine papillomavirus type 2 [62]. Though Hepatitis C virus (HCV) is detected in PBMCs of infected individuals, infected PBMCs are not observed in co-culture with cell culture systems producing HCV virions. This implicates additional reservoirs for the virus and allows for PBMCs infection [63]. However, Polyomavirus BK (BKV), one of the tumor viruses associated with nephropathy in renal allografts, elicits a BKV-specific proliferative response in the PBMCs of healthy individuals and bone marrow transplant recipients [64], [65]. Another human tumor virus, John Cunningham virus (JCV) is detectable in the PMBCs of immunoimpaired and healthy individuals [66]. The well studied EBV infects B lymphocytes and induces their differentiation, proliferation [32]. Furthermore, PBMCs infected EBV lead to immortalization and transformation of B cells [47]. However, the mechanism by which KSHV infects and transforms B cells is not fully understood. Thus, the development of lymphoproliferative diseases which include primary effusion lymphoma and multicentric Castleman's disease is yet to be fully understood. Recently, PBMCs from marmosets orally and intravenously infected with rKSHV.219, showed the presence of the viral genome and LANA expression [42]. Additionally, T and B cells isolated from primary human tonsillar cells were shown to be infected by KSHV virions, although more T cells were infected. However, these infections were abortive without further lytic infection [35], [36]. Other T cells types from human PBMCs can support KSHV infection [67], [68], [69]. Similar patterns were observed showing that both T and B cells from human PBMCs are effectively infected up to 7 days, suggesting that KSHV has a different mode of infection compared EBV infection. Obviously, many more T cells were infected in a time-dependent manner, perhaps due to the large population of T cells or a receptor on T cells not highly expressed in B cells. At 4 and 7 dpi, T cells were infected up to 4.48%, over 2 times the percentage of B cells infected. This phenomenon was also seen in KSHV infected tonsillar cells, though the life span of infected cells was short [36]. This pattern was similar to transformed 293 cells in that RTA_1st_ and RTA_all_ recombinant viruses still enhanced population of T cells in PBMCs, suggesting that infected T cells may be latently infected. Our data also showed that the percentage of CD19 + GFP+ B cells was increased from 1dpi to 7dpi. Unexpectedly, B cell infection boosted the proliferation of infected cells in a time-dependent manner, though T cells are likely to suppress lytic replication of infected B cells. It may be the case that B cells are undergoing lytic cycle replication, releasing progeny which reinfect both T cells and B cells as there was a general increase in lytic cycle gene expression within 2dpi. The population of infected B cells began to decrease at 7dpi, though the number of infected T cells had peaked. As HIV infection may reduce the CD4+ T cell counts, KSHV infected T cells may maintain a fine balance in overall T cell population as well as promote cell proliferation and immortalization for B cells. Another possibility is that certain subpopulations of cells were significantly overactive and may restrict B cell proliferation. Recently, Myoung and Ganem showed that T cells from infected primary human tonsillar lymphoid cells by KSHV did not support proper viral transcription and did not produce infectious virus. However, activated T cells may promote or stabilize latency of KSHV infected B cells [35], [36]. Interestingly, we did not see proliferation of B cells but the percentage of infected B cells was decreased at 7dpi. Therefore we think other restrictive signals are involved in controlling latency of infected B cells, and so transformation for B cells was suppressed. After 7days post-infection, most cells died and no immortalization was observed. One reasonable explanation is that B cells can provide same paracrine signal activities to T cells but infected T cells may lack the ability to receive these signals from B cells, thus losing their proliferative capability. However, B cells may have a central role in infection and proliferation of PBMCs. After 7dpi, the efficiency of infection of B cells was decreased which led to a dramatic reduction in proliferation of the KSHV infected PBMCs. Did the presence of more T cells which were continuously infected destroy the population balance of PBMCs? Do B cells drive essential signaling important for T cells proliferation? These questions merit further investigation. In addition, GFP positive signals showed that more cells were infected in a time-dependent manner. We ruled out the possibility that TPA may have caused this effect in infected T and B cells, as a side-by-side comparison between BAC36 wt, RTA_1st_ and RTA_all_ recombinant viruses strongly supported our conclusion [70]. We clearly showed that RTA_1st_ and RTA_all_ viruses infected total GFP-positive B and T cells showed a prominent increase relative to BAC36 wt in PBMCs up to 7dpi. These results further support our hypothesis that mutation of RBP-Jκ in the RTA promoter can enhance KSHV latent infection of both of B and T cells in PBMCs during primary infection.

The expression of total mRNA from infected PBMCs provides additional information and further reinforces the pattern of KSHV infection. RTA_1st_ and RTA_all_ recombinant viruses showed a decrease in K8 expression, though the down-regulation was not necessarily as strong as in B cells infected with RTA-deficient virus [23]. However, this is reasonable because neither RTA_1st_ nor RTA_all_ recombinant viruses abrogated viral lytic capabilities. RTA_1st_ and RTA_all_ recombinant viruses up-regulated KSHV ORF6 (SSB, single-stranded DNA binding protein) indicating that viral DNA replication is active in infected PBMCs. LANA expression was also up-regulated, suggesting a more tightly latent infection due to the action of LANA on RBP-Jκ. ORF 49 expression was down-regulated in RTA_1st_ and RTA_all_ recombinant viruses infected PBMCs, suggesting that these recombinant viruses may in part lose their lytic capability. Interestingly, the mRNA levels of ORF K8 which is a direct target of RTA [71], was significantly decreased in RTA_1st_ and RTA_all_ recombinant viruses infected PBMCs, strongly suggesting that the two recombinant viruses possess a reduced capability for lytic replication during primary infection. Overall, these data further supports our hypothesis that recombinant RTA_1st_ and RTA_all_ viruses are enhanced in their ability to maintain latency after infection of primary cells.

Though PBMCs were effectively infected, long-term infection of B and T cells was not supported. RTA_1st_ and RTA_all_ recombinant viruses showed on increase in genome copies in long-term-infected TIVE cells providing evidence that mutation of the RBP-Jκ sites within the RTA promoter enhanced KSHV latent infection and induces the proliferative capability of the infected primary cells.

In conclusion, we have now experimentally shown that KSHV recombinant viruses with mutated RBP-Jκ sites within the RTA promoter possess an enhanced ability to maintain latent infection in transformed-293 cells as well as PBMCs during early infection. Our studies further confirm and define our previously published data which showed the effects on truncation of the RTA promoter and has important implications regarding the development of KSHV-associated lymphoproliferative disease. These recombinant viruses now provide a model which can be used to explore the early stages of primary infection in human PBMCs as well as the development of KSHV-associated lymphoproliferative diseases.
